# 1-μm spatial resolution in silicon photon-counting CT detectors

**DOI:** 10.1117/1.JMI.8.6.063501

**Published:** 2021-11-18

**Authors:** Christel Sundberg, Mats Persson, J. Jacob Wikner, Mats Danielsson

**Affiliations:** a KTH Royal Institute of Technology, Department of Physics, Stockholm, Sweden; b Karolinska University Hospital, MedTechLabs, BioClinicum, Solna, Sweden; c Linköping University, Department of Electrical Engineering, Linköping, Sweden

**Keywords:** spatial resolution, silicon detector, charge diffusion, charge transport

## Abstract

**Purpose:** Spatial resolution for current scintillator-based computed tomography (CT) detectors is limited by the pixel size of about 1 mm. Direct conversion photon-counting detector prototypes with silicon- or cadmium-based detector materials have lately demonstrated spatial resolution equivalent to about 0.3 mm. We propose a development of the deep silicon photon-counting detector which will enable a resolution of 1  μm, a substantial improvement compared to the state of the art.

**Approach:** With the deep silicon sensor, it is possible to integrate CMOS electronics and reduce the pixel size at the same time as significant on-sensor data processing capability is introduced. A Gaussian curve can then be fitted to the charge cloud created in each interaction.We evaluate the feasibility of measuring the charge cloud shape of Compton interactions for deep silicon to increase the spatial resolution. By combining a Monte Carlo photon simulation with a charge transport model, we study the charge cloud distributions and induced currents as functions of the interaction position. For a simulated deep silicon detector with a pixel size of 12  μm, we present a method for estimating the interaction position.

**Results:** Using estimations for electronic noise and a lowest threshold of 0.88 keV, we obtain a spatial resolution equivalent to 1.37  μm in the direction parallel to the silicon wafer and 78.28  μm in the direction orthogonal to the wafer.

**Conclusions:** We have presented a simulation study of a deep silicon detector with a pixel size of $12\times 500\;\mu m^{2}$ and a method to estimate the x-ray interaction position with ultra-high resolution. Higher spatial resolution can in general be important to detect smaller details in the image. The very high spatial resolution in one dimension could be a path to a practical implementation of phase contrast imaging in CT.

## 1 Introduction

Photon-counting spectral detectors are predicted to evolve into a new standard for computed tomography (CT), replacing the scintillator-based technology that has been used the last 20 years. In photon-counting spectral CT, each interacting photon results in an electronic pulse that is proportional to the deposited energy and that can be registered using energy thresholds. Individual photons can therefore be counted and registered with respect to energy. This enables higher spatial resolution and improved spectral fidelity, which will translate into higher signal-to-noise ratio in the image.^1^^–^^3^

One of the existing prototype systems for full-field photon-counting CT is based on silicon.^4^ With an edge-on orientation of the silicon wafer, you will get so called “deep silicon,” which has a high detection efficiency. As an industry standard, silicon is a very mature semiconductor material that provides fast charge collection and can be manufactured as almost perfect crystals. The alternative is to use cadmium-based detector materials such as cadmium telluride (CdTe) and cadmium zinc telluride (CZT) that are more challenging to produce. Due to the low atomic number of silicon, another advantage is the absence of K-fluorescence photons, which will limit spectral and spatial resolution for high Z materials. In low Z materials like silicon, there is a significant fraction of Compton interactions, in which the incident photon only deposits some of its energy upon interacting with the detector material.

**Fig. 1:**
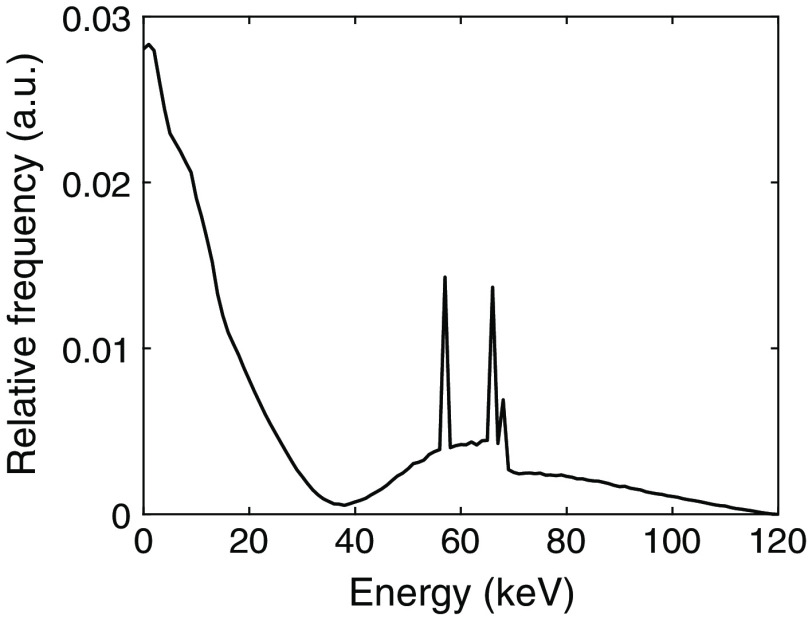
Simulated spectrum of the deposited energies in a silicon detector from an x-ray source operated at 120 kVp including 30 cm soft tissue filtration between the source and the detector.

In Fig. 1, a typical spectrum of the deposited energies in a silicon detector is shown. Compton interactions are visible as the peak between 0 and 35 keV in the spectrum. As there is little to no overlap in energy between photoelectric and Compton events, it is possible to differentiate between the two interaction types. It has been shown that as long as the deposited energy is higher than the lowest threshold of the detector system, a Compton interaction means that a photon is counted and the information is transferred to the image.^5^

Spatial resolution is clinically important in order to resolve small details. Of even more interest, very high spatial resolution in at least one dimension could also be a road forward for clinical implementation of phase contrast imaging. The information retrieved from the analysis of the x-ray phase interference could then be used in combination with the standard absorption image to increase the image quality. This is particularly promising for soft tissue when the absorption contrast is very small. With a very high-resolution detector, the so called $G_{2}$ analyzer grid could be eliminated, which is today a practical obstacle for an implementation of phase contrast imaging.^6^ In a detector with the sensors oriented edge-on to the x-ray beam, the spatial resolution is limited by the pitch between the pixel electrodes and by the wafer thickness. Decreasing the pixel size in any of these directions to achieve extreme spatial resolution is associated with technical challenges such as power consumption since the number of channels is increasing, but at the same time, the capacitance per channel is decreasing, which will mitigate this effect. With emerging CMOS technology, electronics can be integrated onto the silicon sensor material, which enables the pixel pitch to be reduced drastically.^7^^–^^9^

When a photon interacts in a semiconductor detector, electrons and holes are released and initially form a charge cloud. The size of the initial charge cloud increases with the amount of deposited energy. Due to an applied bias voltage, the electrons and holes are transported across the detector and collected by electrodes. The transport consists of both drift, which is caused by the applied voltage and occurs in the direction of the electric field, and diffusion, which is a thermal effect that causes random movement in all directions.

Due to the difference in mobility, electrons and holes travel through the detector at different speeds. It has previously been shown that the interaction position can be determined by measuring the difference in arrival time between the electrons and holes using a double-sided strip detector.^10^ However, this is not possible in detectors with single-sided readout as only one arrival time is registered for each interaction. As the charge carriers drift through the detector, diffusion causes the charge clouds to increase in size. The amount of diffusion depends on the distance between the interaction position and the collecting electrodes. If the charge cloud size is on the same order of magnitude as the pixel pitch or larger, it will give rise to induced currents on several neighboring electrodes. This is commonly referred to as charge sharing. For CT detectors, the pixel pitches normally range between 250 and 1000  μm and charge sharing only occurs for interactions that take place close to pixel borders. Charge sharing then typically causes multiple erroneous events to be registered from a single photon. However, in a system with smaller pixel pitches, it is possible to take advantage of charge sharing. By comparing the induced signals of neighboring pixels, the interaction position can be determined based on the position and size of the detected charge cloud. This allows inferring the interaction position with higher precision than what is possible in the detectors used today.

The interaction position generally becomes more difficult to determine the larger the size of the initial charge cloud. As the charge cloud size depends on the deposited energy, it is easier to determine the interaction position for low-energy interactions. As seen in Fig. 1, for silicon detectors, in contrast to high-Z semiconductor detectors, there are both interactions of high energy, originating from photoelectric interactions, and interactions of low energy, resulting from Compton interactions. The interaction positions for Compton interactions will therefore be easier to determine and are the focus of this work.

A smaller pixel size affects the readout electronics: with smaller electrodes, the capacitance associated with the input amplifier decreases. This can either be exploited to decrease the power consumption and/or to decrease the noise level. The noise level is also affected by the shaping time of the filter component in the readout electronics. By increasing the shaping time it is possible to decrease the noise, but this comes at the expense of increased pulse length, which reduces the count-rate capability. However, with smaller pixels, the number of incident photons per pixel decreases and the demand on high count rate capability is reduced. This allows using a longer shaping time to reduce the noise level without altering the count-rate performance.

Techniques, in which the spatial resolution is improved by utilizing information from a collection of detector elements, are common in nuclear medicine.^11^ In a detector involving photomultiplier tubes, an interaction position can be obtained as a mean position based on the signal strength and position of each photomultiplier. For semiconductor x-ray detectors in general, similar approaches have been reported involving weighted sums.^12^ In detectors for free electron laser x-ray sources, subpixel resolution can be obtained using interpolation algorithms.^13^^,^^14^ Among imaging application specific integrated circuits (ASICs), charge summing concepts have been exploited to utilize information from multiple pixels in order to determine an interaction position.^15^^,^^16^

In this work, we present a simulation study in which a silicon detector for CT with a pixel size of $500\times 12\;\mu m^{2}$ is investigated. By combining a Monte Carlo photon simulation with a charge transport model, we study the charge cloud distributions and induced currents as functions of the interaction position. From the simulated pixel responses to interactions at different detector positions, we propose a method to estimate the interaction position and present the resulting point spread function (PSF) and corresponding modulation transfer function (MTF).

## 2 Method

### 2.1 Detector Geometry

**Fig. 2:**
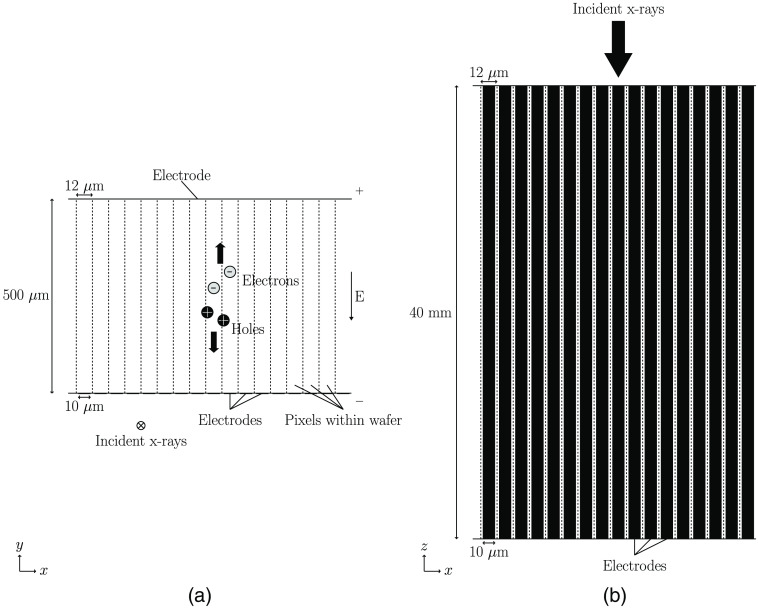
(a) Simulated detector geometry as seen from the perspective of an x-ray source. The detector consists of a 500-μm thick silicon wafer. In the x direction, 51 electrodes are positioned along one side of the wafer. The width of each electrode is 10  μm and the pixel pitch is 12  μm. A single electrode is found at the opposite side of the wafer. A bias voltage is applied in the y direction, resulting in an electric field that points in the negative y direction. The incident x-rays enter the detector in the negative z direction. An example of released charge carriers is included and shows the drift direction of electrons and holes, respectively. (b) Simulated detector geometry as seen from the side of the wafer. Note that the images are not to scale.

In this work, an edge-on silicon detector was simulated as a silicon wafer of thickness 500  μm with a pixel pitch of 12  μm and an electrode size of 10  μm. In total, 51 pixels were included in the simulation, ensuring the collection of all released charge carriers. In Fig. 2, the detector geometry is shown along with the definition of the x and y directions used in this work. A potential difference between the front side electrodes and the backside electrode was obtained by simulating a voltage of 200 V applied to the backside electrode.

### 2.2 Electron Tracks

To obtain the initial charge cloud, Compton interactions between incident x-ray photons and the detector material were simulated using PENELOPE, a Monte Carlo simulation package that simulates electron–photon showers in the range of 50 eV to 100 MeV.^17^ In a Compton interaction, an electron is scattered by an incident photon. The scattered electron travels through the detector material and deposits energy through a series of inelastic collisions and inner-shell ionizations, creating an electron track. In PENELOPE, the interactions and resulting secondary particles are recorded along with the deposited energy and corresponding coordinates. From the resulting electron track, the energy deposited at each position was converted into electron–hole pairs by dividing the deposited energy with the silicon ionization energy of 3.6 eV.^18^

A total of 100,000 Compton interactions were simulated based on incident photons of energies sampled from the spectrum of an x-ray source operated at 140 kVp filtered with 8.48 mm aluminum, 0.8 mm beryllium, and 30 cm soft tissue. The initial interaction position $\left(X_{0},Y_{0}\right)$ of each incident photon was randomly selected within the $12\times 500\;\mu m^{2}$ pixel area of pixel 26, equidistant from the edges of the wafer. In this work, only primary Compton interactions were considered, i.e., primary photoelectric events were not included in the studied set of interactions. Secondary photon interactions arising from Compton scattered photons have a high probability of escaping from the wafer and can be blocked by tungsten sheets between wafers and were therefore not considered.^5^

### 2.3 Charge Transport and Signal Induction

In a semiconductor detector, the released electron–hole pairs are separated immediately after generation due to the applied electric field. The electric field causes the charge carriers to drift in opposite directions with a velocity, $\bar{v_{d}}$, given by

$$\bar{v_{d}}=\mu \bar{E},$$ (1)

where μ is the charge carrier mobility and $\bar{E}$ is the electric field. Based on the small pixel size in relation to the thickness of the wafer, the bending of the electric field lines was considered to be negligible and a uniform electric field was assumed throughout the wafer. This results in a drift velocity that is parallel to the y direction. In this work, a field-dependent mobility was used for the drift velocity.^19^

Diffusion causes random movement in all directions and enlarges the charge cloud. To simulate diffusion, we assumed a diffusion coefficient D that is expressed by the Einstein law:

$$D=\frac{kT}{e}\mu ,$$ (2)

where k is the Boltzmann constant, T is the absolute temperature, and e is the fundamental electron charge. For this case, the mobility was assumed to be constant. Based on the electric field strength, this assumption is in good agreement with experimental results.^19^

In this work, charge movement was considered for a two-dimensional case assuming that the electrodes are long in the z direction and that nonuniformities in z can therefore be ignored. For each charge carrier, the diffusion distance dx in the direction of x was obtained by sampling from the Gaussian probability distribution P(x) expressed as

$$P(x)=\frac{1}{\sqrt{4\pi Ddt}}\;\exp(-\frac{x^{2}}{4Ddt}),$$ (3)

where dt is the time step of the diffusion. In this work, dt was set to 2 ns. Similarly, the diffusion distance in y was sampled separately from the same distribution. From this, the resulting total velocity $\bar{v}$ of each charge carrier becomes

$$\overset{¯}{v}=\overset{¯}{v_{d}}+\overset{¯}{v_{D}},$$ (4)

where $\bar{v_{D}}$ describes the velocity caused by diffusion according to $\bar{v_{D}}=(dx/dt,dy/dt)$. In our current simulation framework, Coulomb repulsion is not included.

Due to the movement of charge, the detector electrodes measure induced currents. To calculate the induced current i(t) on each electrode, the Shockley–Ramo theorem was used^20^^,^^21^

$$i(t)=e{\overset{¯}{E}}_{w}(x,y)\overset{¯}{v}(t),$$ (5)

where ${\overset{¯}{E}}_{w}(x,y)$ is the weighting field for a charge carrier in position (x,y), $\bar{v}$ is the carrier velocity, and t is the drift time. For each electrode, the weighting potential W was calculated by solving the Laplace equation in two dimensions for a system, in which the electrode was set to unit potential and all other electrodes were set to a zero potential. The weighting field was then obtained as ${\bar{E}}_{w}(x,y)=-\nabla W$.

Throughout all simulations of charge transport, a silicon resistivity of 15  kΩ·cm was used. Furthermore, a high purity of the detector material was assumed and trapping effects were therefore excluded.

### 2.4 Simulated Noise

To simulate the effect of electronic noise, a noise level of σ=0.22  keV was assumed. For each pixel, this was incorporated by adding Gaussian white noise with a standard deviation of σ to the total current detected by the pixel. No correlation of the noise between pixels was assumed. In silicon detectors, it is desirable to detect Compton interactions down to 0 keV. However, there is a trade-off between detecting as many interactions as possible and minimizing the number of noise counts. Based on the noise level in this work, a lowest threshold of 4σ=0.88  keV was therefore set. For a readout system in which the signal is sampled every 10 ns, this corresponds to one noise count every 150  μs.

### 2.5 Estimation of Interaction Position

In a photon-counting detector system, the induced current measured by each pixel is processed using readout electronics. This results in an electronic pulse with a pulse height that corresponds to the collected charge and thereby the interaction energy. The pulse height is typically sampled at regular time intervals using energy thresholds. In this work, for simplicity, the readout electronics were not simulated. Instead, the total induced current was used directly to represent the energy measured by each pixel.

For each simulated Compton interaction, the total induced current in each of the 51 pixels was extracted as the integral of i(t) in Eq. (5). To obtain an estimate of the charge distribution upon collection, a Gaussian curve fit of the form $f(x)=ae^{-\;\frac{{\left(x-b\right)}^{2}}{2c^{2}}}$ was performed based on the measured current among the pixels. For this, it was assumed that the total current in each pixel was deposited at the x direction center of the pixel. Since we expect diffusion to increase the charge cloud size symmetrically around the initial electron track, the initial interaction position in x can be estimated based on the center of mass of the charge distribution. With the Gaussian curve fit, this corresponds to the parameter b, which indicates the maximum position of the Gaussian curve. Correspondingly, due to diffusion, we also expect the charge cloud size to increase with increasing drift time. The charge distribution upon collection should therefore be wider for interactions that occur far from the collecting electrodes. To estimate the interaction position in the y direction, we therefore use the Gaussian parameter c, which determines the Gaussian curve width. Overall, it is important to note that the fitted Gaussian curve is used to estimate the shape of the charge distribution and that it does not relate to the collected charge or deposited energy, i.e., the maximum value of the Gaussian curve is not an indication of the interaction energy.

For each interaction, based on the fitted Gaussian, the resulting value of b then directly corresponds to the interaction position in x. The resulting value of c is the estimated charge cloud width and relates to the amount of charge diffusion and thereby to the interaction position in y. However, to determine the interaction position in y, the relation between the estimated charge cloud width c and depth of interaction y, $c_{\text{fit}}(y)$, must first be known. This was obtained by studying the relation between the obtained values of c and the true interaction positions in y for the entire dataset.

With the estimated interaction position in x defined as $X_{e}=b$ and the resulting estimated position in y, $Y_{e}$, obtained via $c_{\text{fit}}(y)$, an estimated interaction position was determined for each simulated interaction. Based on the detector geometry, $Y_{e}$ was allowed a maximum value of $Y_{e,\max}=500\;\mu m$ and a minimum value of $Y_{e,\min}=0\;\mu m$. Since we only evaluate a single interaction at a time, for the case with no electronic noise, no limits were used for the parameters in the Gaussian fit. However, for the case with included electronic noise and a lowest threshold, it was found that each interaction was measured by a maximum of five pixels. Since all interactions were simulated in pixel 26, five pixels correspond to the interval [−30,30]  μm from the center of pixel 26. Any nonzero signal found outside this interval therefore arises from electronic noise. In order to prevent Gaussian fits to obvious noise counts in pixels far away, the parameter b was limited to the interval [−30,30]  μm.

To facilitate position estimation for interactions where the estimated charge cloud width is below $c_{\text{fit}}(0)$ or above $c_{\text{fit}}(500)$, the mean interaction position was calculated for each such case: ${\overset{¯}{y}}_{c<c_{\text{fit}}(0)}$ and ${\overset{¯}{y}}_{c>c_{\text{fit}}(500)}$. Based on this, the position estimation was implemented according to Algorithm 1.

**Algorithm 1:** Algorithm for position estimation.

Fit Gaussian function $f(x)=ae^{-\;\frac{{\left(x-b\right)}^{2}}{2c^{2}}}$ to total induced currents in pixels
$X_{e}=b$
**if** $c<c_{\text{fit}}(0)$ **then**
$Y_{e}={\overset{¯}{y}}_{c<c_{\mathrm{fit}}(0)}$
**else if** $c>c_{\mathrm{fit}}(500)$
$Y_{e}={\overset{¯}{y}}_{c>c_{\text{fit}}(500)}$
**Else**
Find $Y_{e}$ such that $c=c_{\text{fit}}(Y_{e})$ is satisfied
**End**

The interaction position estimation was first performed for the dataset of simulated interactions without simulated noise and with no lowest threshold. This represents an ideal case in which even small pixel currents contribute to the estimation. In a second evaluation round, corresponding to a more realistic implementation, the same dataset was processed but including electronic noise and a lowest threshold. When applying the lowest threshold, pixels with detected energies below the threshold were set to zero in the Gaussian fit. The estimated positions were in both cases compared to the ground truth interaction positions $\left(X_{0},Y_{0}\right)$ in order to analyze the accuracy of the estimation.

## 3 Results

**Fig. 3:**
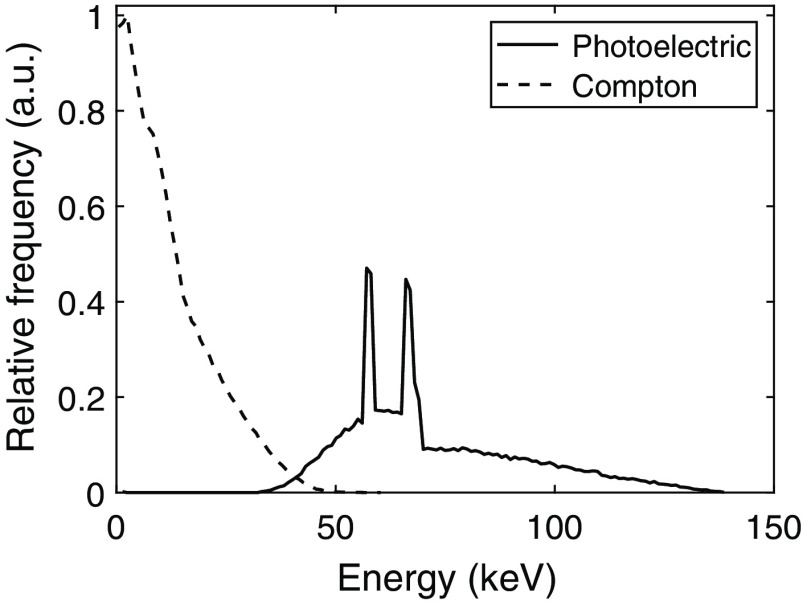
Spectrum of the deposited energies in the simulated silicon detector as obtained from PENELOPE. The incident photons were sampled from the spectrum of an x-ray source operated at 140 kVp and filtrated with 8.48 mm Al, 0.8 mm Be, and 30 cm soft tissue. Photoelectric events are plotted separately. In this work, interaction positions were estimated for Compton interactions only.

The spectrum of simulated Compton interactions is shown in Fig. 3 along with the spectrum of primary photoelectric interactions that were excluded from the analysis.

**Fig. 4:**
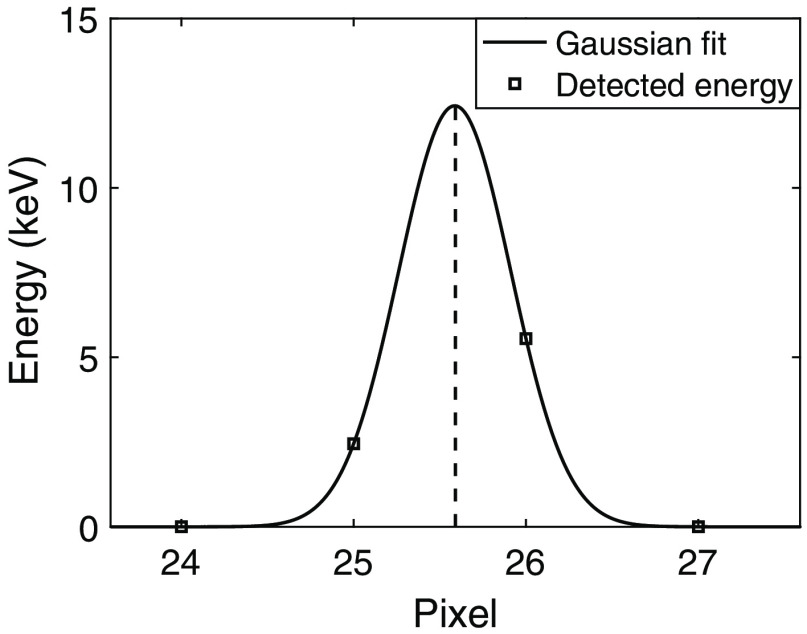
Example of an interaction of 8 keV in position $X_{0}=-4.8\;\mu m$ (with x=0 corresponding to the middle of pixel 26) and $Y_{0}=20\;\mu m$ as detected by the simulated pixels. No noise was included. A Gaussian curve was fitted to the energy detected by each pixel. The estimated interaction position in x, $X_{e}$, is given by the position of the maximum of the Gaussian, which is indicated by the dashed line. Note that the fitted Gaussian curve is used to facilitate estimation of the interaction position and does not relate to the deposited energy itself, i.e., the maximum value of the Gaussian curve is not an indication of the interaction energy.

An example of a Gaussian curve fit to the energies detected by the pixels is presented in Fig. 4. The detected energies result from an interaction of 8 keV for which $Y_{0}=20\;\mu m$, with the front side electrode defined as y=0, and $X_{0}=-4.8\;\mu m$, with x=0 corresponding to the middle of pixel 26. The estimated position $X_{e}$ is obtained from the position of the maximum of the fitted Gaussian curve.

**Fig. 5:**
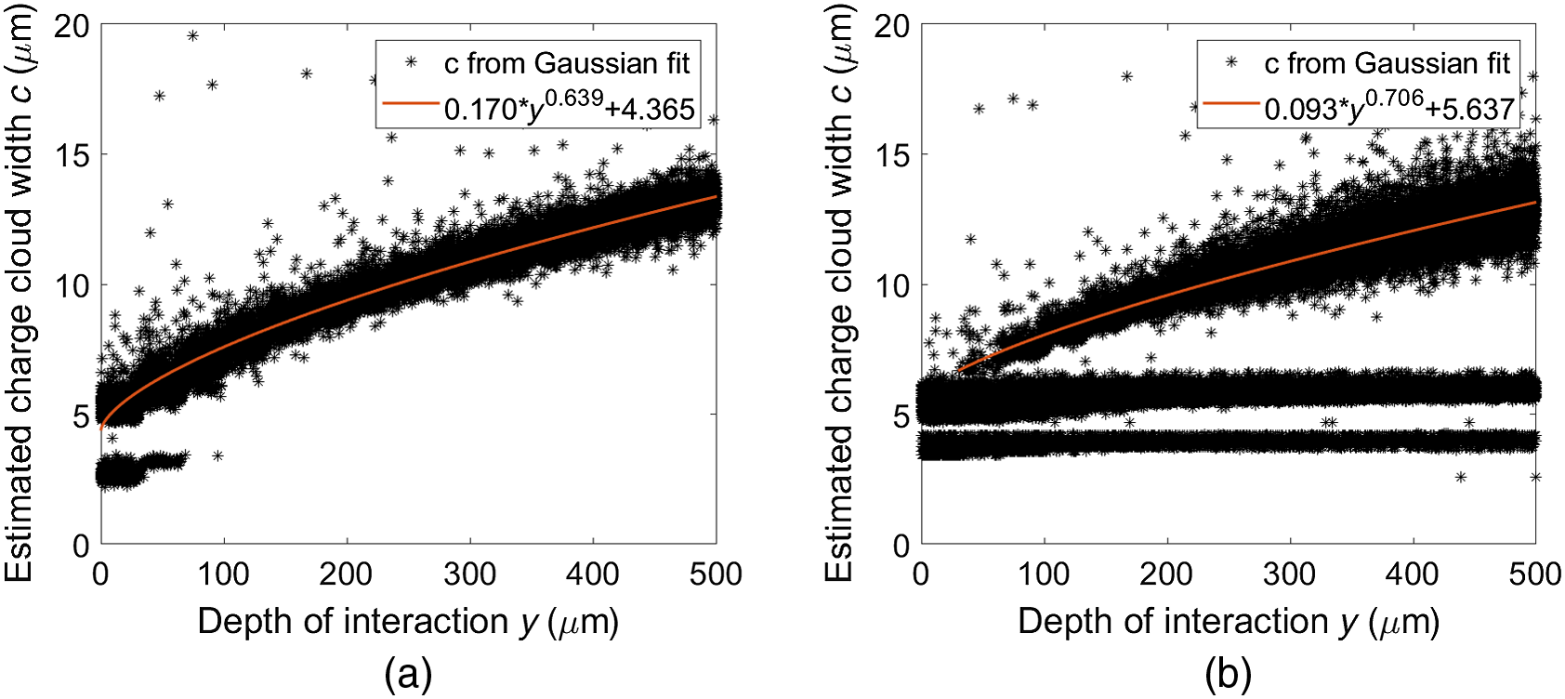
Scatter plots of the estimated charge cloud width c for each interaction position in y. (a) Estimated charge cloud widths obtained from Gaussian fits to pixel responses from the ideal dataset. (b) Estimated charge cloud widths obtained from Gaussian fits to pixel responses from the dataset including noise and a lowest threshold. For each dataset, a power function was fitted to the estimated charge cloud widths to obtain $c_{\text{fit}}(y)$, which was used to estimate the interaction position in y, $Y_{e}$.

In Fig. 5, the scatter plot of the estimated charge cloud width c is shown along with the obtained relation of $c_{\text{fit}}(y)$ for each of the two datasets. For the case without noise, estimated charge cloud widths below 4  μm were excluded in the curve fit. Correspondingly, for the case with noise and a lowest threshold, charge cloud widths below 6.6  μm were excluded. For simplicity, the mapping from c was assumed to be independent of interaction energy throughout. For the case without noise, the mean interaction positions for estimated charge cloud widths outside the range of $c_{\text{fit}}$ resulted in ${\overset{¯}{y}}_{c<c_{\text{fit}}(0)}=17\;\mu m$ and ${\overset{¯}{y}}_{c>c_{\text{fit}}(500)}=479\;\mu m$. For the case with noise, the corresponding mean values were ${\overset{¯}{y}}_{c<c_{\text{fit}}(0)}=187\;\mu m$ and ${\overset{¯}{y}}_{c>c_{\mathrm{fit}}(500)}=463\;\mu m$.

**Fig. 6:**
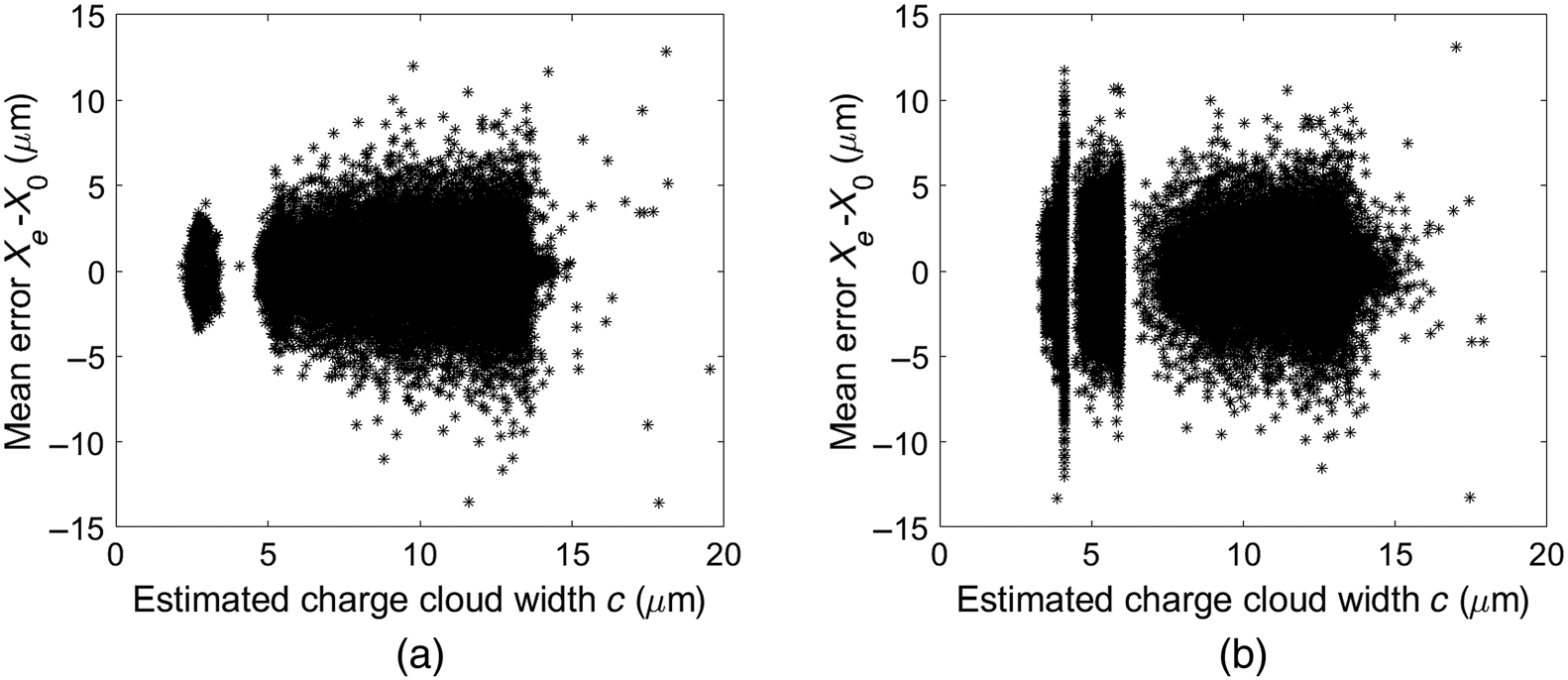
Scatter plots of the error in the estimated position in x, $X_{e}-X_{0}$, versus the estimated charge cloud width c. (a) Error in estimated position in x versus the charge cloud widths obtained from Gaussian fits to pixel responses from the ideal dataset. (b) Error in estimated position in x versus the charge cloud widths obtained from Gaussian fits to pixel responses from the dataset including noise and a lowest threshold.

Table 1 shows the calculated errors between the estimated interaction positions $\left(X_{e},Y_{e}\right)$ and the true interaction positions $\left(X_{0},Y_{0}\right)$ for each dataset. In Fig. 6, we show the correlation between the errors in the estimated x positions and the parameter c which is used to determine the interaction position in the y direction.

**Table 1:** Mean absolute error and standard deviation of the estimated interaction positions for each dataset.

Dataset	Ideal, σ=0 keV, no threshold	σ=0.22 keV, threshold of 4σ
Mean absolute error $\left|X_{e}-X_{0}\right|$ (μm)	0.65	1.37
RMS error of $X_{e}-X_{0}$ (μm)	1.09	1.87
Mean absolute error $\left|Y_{e}-Y_{0}\right|$ (μm)	12.79	78.28
RMS error of $Y_{e}-Y_{0}$ (μm)	18.28	106.44

**Fig. 7:**
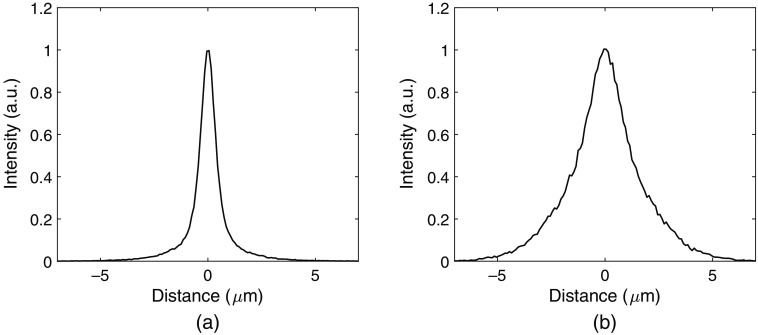
PSF of the detector and the proposed method of interaction position estimation in the x direction. (a) Ideal data with no electronic noise or lowest threshold. (b) Electronic noise and a lowest threshold of 0.88 keV included.

**Fig. 8:**
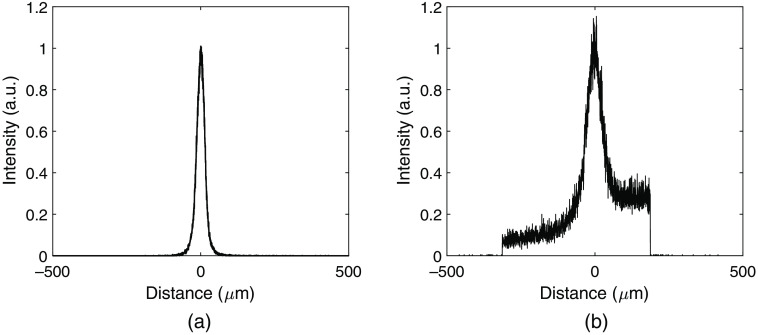
PSF of the detector and the proposed method of interaction position estimation in the y direction. (a) Ideal data with no electronic noise or lowest threshold. (b) Electronic noise and a lowest threshold of 0.88 keV included.

The resulting PSF for the detector and the proposed method for estimating the interaction position is shown in Fig. 7 for x and in Fig. 8 for y. The PSFs were obtained as the scaled histograms of the differences between the true and estimated positions for all simulated events. A Gaussian curve fit was performed to each PSF resulting in a full-width at half-maximum (FWHM) of 0.84  μm for x and 30.75  μm for y with the ideal dataset. For the dataset including noise, the corresponding FWHM values were 2.50  μm and 71.30  μm for directions x and y, respectively.

**Fig. 9:**
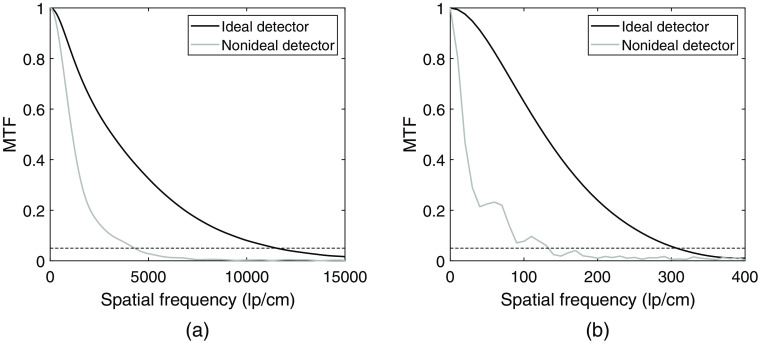
MTFs of the detector and the proposed method of interaction position estimation. The ideal detector includes no electronic noise or lowest threshold. For the nonideal detector, electronic noise and a lowest threshold of 0.88 keV are included. The dashed line indicates 5% of the MTF. (a) MTF in the x direction and (b) MTF in the y direction.

Figure 9 shows the MTFs of the detector and the proposed method obtained as the absolute Fourier transform of the presented PSFs. At 5% of the MTF, the ideal detector with no electronic noise can resolve $1.15\times 10^{4}\;\mathrm{lp}/\mathrm{cm}$ in the x direction and 307  lp/cm in the y direction. For the case with electronic noise and a lowest threshold, the corresponding values are 4294  lp/cm and 133  lp/cm, respectively.

## 4 Discussion

As seen in Fig. 3, the spectrum obtained from the PENELOPE simulations corresponds well to the spectrum presented in Fig. 1. Based on the simulated spectrum, Compton interactions constitute 66.2% of all primary interactions. With a lowest threshold of 0.88 keV, ∼5% of the Compton interactions are below the threshold. With the lowest threshold included, the estimation of interaction position thereby affects the spatial resolution of 63% of the registered interactions.

Regarding the interaction position estimation, the fitted curves in Fig. 5 show a similar behavior for the ideal dataset and the dataset with included noise. For the ideal dataset in Fig. 5(a), a collection of low values of the estimated charge cloud width c is seen between wafer depths of 0 and 75  μm. For interactions that occur close to the collecting electrodes, the effects of diffusion are small and the charges that contribute to the induced signals are almost entirely collected by a single pixel. For such cases, the Gaussian fit becomes based on practically one nonzero pixel value, resulting in a narrow fitted curve. Outliers that show high values of the estimated charge cloud width are caused by fluorescence events. For silicon, fluorescence edges are found at ∼1.75  keV, which is typically below the lowest threshold. However, in a system of lower noise, such as the detector in this study, these events will be registered. If a fluorescence event occurs, the resulting electron track consists of two separate parts: one track starting at the initial interaction position and one track from the interaction of the fluorescence photon at a different interaction position. This causes a spread in the distribution of induced current and results in an erroneous Gaussian curve fit. This type of error could be avoided by setting upper limits to the fitted Gaussian curve or by implementing an anti-coincidence logic that can separate or correct for the fluorescence events. It is important to note that it is possible to identify all fluorescence photons based on energy, as these will always deposit ∼1.75  keV upon interacting with the detector material.

For the dataset including noise, the Gaussian fit results in many low values of the estimated charge cloud width around 5  μm. These can be seen as the horizontal lines in Fig. 5(b). Similar to the ideal dataset, these values arise from Gaussian fits in which one pixel has collected almost all charge. The lowest horizontal line represents cases in which the Gaussian fit is entirely based on a single nonzero pixel, whereas the horizontal line above it corresponds to two or three nonzero pixels of which one pixel is typically dominant. Since the measured pixel value is set to zero in all pixels where the induced current is below the threshold, the fitted Gaussian curve becomes narrower for cases when data points have been lost below the threshold. Since the low estimated charge cloud widths occur for all interaction positions in y, the resulting error in the estimated interaction position increases compared to the case with no noise.

From Table 1, it is clear that the performance of estimating the position in y largely decreases when noise and a lowest threshold are included. This is directly caused by interactions for which the estimated charge cloud widths cannot be mapped to the fitted curve $c_{\text{fit}}$ in order to estimate $Y_{e}$. The estimated interaction position in x, on the other hand, is less affected by noise and the applied threshold. The average absolute error $\left|X_{e}-X_{0}\right|$ increases with a factor of 2.11 when noise is included, but remains below 1.5  μm.

It can be seen from Fig. 6 that there is little correlation between the errors in estimated position for the x direction and the parameter c that is used to estimate the interaction position in the y direction. Similar to Fig. 5(a), the scatter plot for the ideal dataset exhibits a cluster of values for c<4.8  μm. This cluster corresponds to charges that are almost entirely collected by a single pixel, which typically occurs when the interaction position is located centrally in the pixel and close to the collecting electrodes. The estimated position then becomes the center of pixel 26 (the illuminated pixel) and the error between the actual position and the estimated position becomes small. Errors in the estimated x position appear to increase slightly with an increasing value of the parameter c. For the case with electronic noise and a lowest threshold, Fig. 6(b) has two distinct lines for low values of the parameter c. These correspond to the two horizontal lines seen in Fig. 5(b) that occur when one pixel collects most of the charge. For such cases, the resulting errors in the estimated x position are larger compared to the ideal case with no electronic noise and no lowest threshold. For the ideal case, the maximum charge is typically collected by pixel 26. However, with electronic noise, the highest signal sometimes occurs in one of the neighboring pixels which results in an estimated interaction position $X_{e}$ that is outside the illuminated pixel 26 and therefore more erroneous. Overall, the ability to estimate the position in x does not appear to depend substantially on the parameter c.

The presented point-spread functions for x in Fig. 7 are both smooth and symmetric, with mean errors of zero. From this, it can be concluded that there are no obvious systematic errors in the position estimation. As the errors increase for the dataset with added noise, the corresponding PSF becomes wider. The resulting FWHM is a factor of three higher for the dataset including noise, which is expected due to the higher average absolute errors and the higher RMS errors. The point-spread functions in y, presented in Fig. 8, show a larger difference compared to the PSF curves for x. For the ideal dataset, the PSF is symmetric and has a zero mean error, similar to the PSFs for x. However, for the dataset with noise, the PSF is asymmetric with sharp cut-offs at −313(=187−500)  μm and 187  μm. This is again caused by interactions that cannot be mapped according to the fitted curve $c_{\text{fit}}$ that instead become mapped directly to $Y_{e}={\overset{¯}{y}}_{c<c_{\text{fit}}(0)}=187\;\mu m$. The mapping to $Y_{e}={\bar{y}}_{c>c_{\mathrm{fit}}(500)}=493\;\mu m$ is in comparison not very frequent and results in smaller errors which are therefore not as visible in the PSF.

The MTFs in Fig. 9 show a clear difference between the ideal case with no noise and the case with electronic noise and a lowest threshold. At 5% of the MTF, the resolution for the no noise case is 2.7 times higher in x and 2.3 times higher in y compared to the noise case.

With the presented method, the estimation of the interaction position in x is robust and highly accurate. However, to improve the presented method further, it is desirable to improve the position estimation in the y direction. We expect this to require a more sophisticated estimation method. Improved results could possibly be obtained by including the interaction energy in the estimation. A relation between the total energy and the distribution of charge could then be exploited to potentially improve the accuracy of the performed curve fits.

In this work, the noise level of σ=0.22  keV was used as a rough estimate. It is based on a previously reported noise level of σ=1.36  keV for a detector with a 125-μm electrode width^22^ that was rescaled according to electrode width and expected incident count-rate per electrode assuming a constant power consumption per area unit. The resulting estimated value of σ=0.22  keV corresponds well to previously reported noise levels for similar detector geometries.^6^^,^^23^ However, to obtain a more accurate noise estimate, the total electrode size, the distance between electrodes, as well as the ASIC design should be considered. For example, if the electrodes are very long in the direction of the incident photons, we expect the noise level to increase. With the presented method, we expect an increased noise level to be most detrimental for position estimation in the y direction. For the x direction, on the other hand, as long as the energy is above the lowest threshold, we expect the spatial resolution in x to be in the order of the pixel pitch.

The simulation of charge transport does currently not include the effect of Coulombic repulsion. This effect is largest at the beginning of the charge transport, when charge carriers are close to each other. In our simulation software, charges are tracked individually. This causes the computational complexity to increase substantially if Coulombic forces are included. There have been many previous studies that have described the effect of Coulomb repulsion on the charge cloud size in silicon detectors.^24^^,^^25^ Based on results by Castoldi et al.,^26^ we expect the charge cloud size to typically be 10% to 15% larger compared to the simulated charge clouds in this work. However, for interactions of higher energies that occur close to the collecting electrodes, the effect could be more than 30%.

Since energy resolution was only considered from the perspective of electronic noise in this work, the number of energy bins as well as time resolution could affect the performance of the presented method. In a practical implementation, it is desirable to have energy bins that are comparable to or smaller than the electronic noise amplitude. With a maximum photon energy of 140 keV, an average bin size of ∼0.5  keV corresponds to 256 energy levels, which is straightforward to implement using an analog-to-digital converter. Based on a previously presented model of an analog readout channel, we expect that a digital peak detection method with a sampling interval of 10 ns will be able to determine the pulse height with an error smaller than 0.1 keV for Compton energies.^27^

Although the presented method was evaluated for Compton interactions only in this work, the method is independent of interaction type and could also be applied to low-energy photoelectric interactions. The main limitation is the electron track length which increases with increasing energy. To enable the estimation of interaction position for interactions of higher energies, there are a number of obstacles to overcome. For example, for a photoelectric event of 70 keV, the average size of the initial charge cloud is 14  μm. However, the initial charge clouds are randomly distributed in relation to the detector geometry with largely varying lengths in x and y. This inherent dispersion of the charge cloud length in x causes interactions of the same energy and same interaction position to be measured by a different number of pixels. For high interaction energies with initial charge cloud sizes on the same order of magnitude as the pixel size, the effect of diffusion is smaller than the variations caused by the initial distribution of the charge cloud. This poses a problem with the proposed method when estimating the interaction position in x and y. In a practical implementation, using a photon-counting spectral detector system, we expect to be able to differentiate between high- and low-energy interactions using specific energy thresholds. It could thereby be possible to create an ultra-high-resolution image based on only low-energy interactions. Correspondingly, an image with lower spatial resolution could be obtained using interactions of higher energies. We see the potential in combining information from both the low-energy and high-energy counts into one image using different weighting schemes. It will be a future question how to best combine the phase contrast image, obtained primarily from the high-resolution image of Compton interactions, with the absorption image, which will be based on both Compton and photoelectric interactions.

The spatial resolution that we obtain in the x direction is on the same order of magnitude as a previously reported detector that has been evaluated for single-shot grating-based phase contrast imaging.^6^ We therefore see the potential of using the detector and proposed method for such applications. For applications in CT, the focal spot size is typically much larger than the presented spatial resolution and therefore limits the potential benefit of a high-resolution detector. However, since the tendency in x-ray source development is toward smaller and smaller focal spot sizes, this might be less of a problem in the future. In grating-based phase contrast imaging, images can be obtained with a conventional x-ray tube by employing a source ($G_{0}$) grating to obtain a spatially coherent x-ray beam despite having an extended focal spot.^28^

In future work, it is desirable to investigate the proposed method for different detector geometries involving both larger and smaller pixel pitches and electrode widths. It is also of interest to develop more sophisticated algorithms to estimate the interaction position. One way of doing this could be using machine learning to estimate the interaction position based on a learned relation between the interaction position and resulting pixel response.

## 5 Conclusions

We have presented a simulation study of a deep silicon detector with a pixel size of $12\times 500\;\mu m^{2}$ and a method to estimate the x-ray interaction position with ultra-high resolution. With realistic electronic noise and a minimum threshold of 880 eV, an error of 1.37  μm was obtained in the direction along the silicon wafer and 78.28  μm in the direction orthogonal to the silicon wafer. The very high resolution obtained in one dimension is of particular interest since it can facilitate x-ray phase contrast imaging in a future CT scanner. It would imply that the analyzer grating used after the patient to measure the interference pattern could be omitted. As proven before, a standard x-ray tube could be used if coherence is improved with a dedicated so called $G_{0}$ grating. Further work involves the development of more sophisticated algorithms that can be used to improve the estimation of interaction positions and the investigation of other detector geometries. We also look forward to building and measuring on real prototype ultra-high resolution deep silicon detectors.
